# Isolation of Rhizoma Paridis saponins as novel entry inhibitors of Crimean-Congo hemorrhagic fever virus

**DOI:** 10.1016/j.cellin.2026.100328

**Published:** 2026-04-28

**Authors:** Ruikun Du, Chen Liu, Xi Wang, Huiying Hou, Xu Wang, Zhaoyu Chen, Jazmin Galvan Achi, Qinghua Cui, Lijun Rong, Manli Wang, Rong Rong

**Affiliations:** a Qingdao Academy of Chinese Medical Sciences, Shandong University of Traditional Chinese Medicine, Qingdao, 266041, Shandong, China; b College of Pharmacy, Shandong University of Traditional Chinese Medicine, Jinan, 250355, Shandong, China; c State Key Laboratory of Virology and Biosafety, Wuhan Institute of Virology, Center for Biosafety Mega-Science, Chinese Academy of Sciences, Wuhan, 430071, Hubei, China; d Department of Microbiology and Immunology, University of Illinois at Chicago, Chicago, IL, 60612, USA

**Keywords:** Crimean-Congo hemorrhagic fever virus, Viral entry inhibitor, Rhizoma paridis, Saponin

## Abstract

Crimean-Congo hemorrhagic fever virus (CCHFV) is a highly pathogenic bunyavirus, causing Crimean-Congo hemorrhagic fever (CCHF) in humans with high morbidity and mortality. Currently, there are no licensed antiviral drugs or vaccines against CCHFV, emphasizing the critical need to develop novel antiviral agents. The entry process is the initial step during the viral life cycle, and has provided an attractive target for novel antiviral development. In the present study, we developed a high-throughput screening approach based on the CCHFV glycoprotein (Gn/Gc)-based HIV-1 pseudoviruses, in order to discover novel CCHFV entry inhibitors. As a pilot, a library consisting of 500 pre-purified fractions of traditional Chinese medicine was screened, and it was identified that multi-fractions of Rhizoma Paridis exhibited inhibitory effects against CCHFV entry. Next, bioactivity-guided isolation was performed and a range of Rhizoma Paridis saponins were characterized as potential anti-CCHFV actives. Moreover, the antiviral activities of both Rhizoma Paridis extracts and the saponin derivatives were validated by a cell-based assay using authentic viruses. Mechanistic studies further demonstrated that Rhizoma Paridis saponins act by targeting host factors. In summary, this study provides a promising new strategy for CCHF treatment that deserves further development in the future.

## Highlights

• Rhizoma Paridis extract shows inhibitory effect against entry of Crimean-Congo hemorrhagic fever virus (CCHFV).
• Rhizoma Paridis saponins are the major active components against CCHFV.
• The saponin actives of Rhizoma Paridis act against CCHFV by interfering with viral entry-associated host factors.

## Graphical abstract

**Figure:**
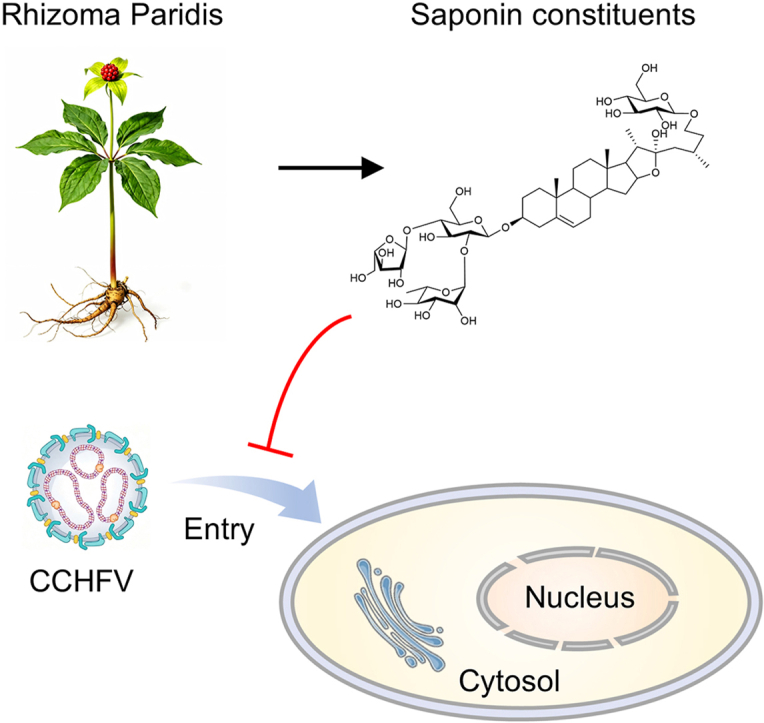

## 1 Introduction

Crimean-Congo hemorrhagic fever virus (CCHFV) is a biosafety level-4 (BSL-4) pathogen that causes severe acute infectious disease in humans called Crimean-Congo hemorrhagic fever (CCHF), with case fatality rates of up to 82% (Rao et al., 2026). Since its first recognition in the 1940s, CCHF has spread widely to about 50 countries throughout Africa, Asia, and Europe. In addition, more than 1 000 cases are reported worldwide annually (Hoogstraal, 1979; Nasirian, 2020). Due to the lack of effective vaccines and drugs and the potential to cause a public health emergency, CCHF has been listed by World Health Organization (WHO) as one of the top priority diseases needing urgent research and development (Allayeh et al., 2025). All these emphasize the urgent demand for developing efficient antivirals against CCHFV infection.

CCHFV belongs to the *Orthonairovirus* genus in *Nairoviridae* family of *Bunyavirales* order.

The viral RNA genome is single-stranded, negative-sense and comprises tripartite segments termed large (L), middle (M) and small (S). These encode the RNA-dependent RNA polymerase (RdRp), glycoprotein precursor (GPC) and nucleoprotein (NP), respectively (Bente et al., 2013). The M-encoded GPC is co-translationally cleaved by cellular proteases to generate two structural glycoproteins (GPs), Gn and Gc, and three non-structural proteins Mucin, GP38 and NSm (Bergeron et al., 2007). The Gn and Gc heterodimers form a locally ordered lattice on the mature virion surface, which are responsible for the virus entry process, including both receptor binding and subsequent fusion of the viral envelope with host cellular membranes (Li et al., 2022; Mishra et al., 2022).

Virus entry into the host cell is the first step of viral replication cycle, and has become one of the most attractive targets for developing novel antiviral agents (Su et al., 2022). To date, numerous entry inhibitors of diverse viruses have been discovered (Dong et al., 2024; Du et al., 2022; Zhu et al., 2023), with some having been approved, such as Enfuvirtide, Albuvirtide, Fostemsavir and Maraviroc (HIV-1 entry inhibitors) (Fadel & Temesgen, 2007; Lalezari et al., 2003; Markham, 2020; Su et al., 2020), Umifenovir (Influenza virus entry inhibitor) (Kadam & Wilson, 2017), as well as various neutralizing antibody-based therapeutics (Otsubo & Yasui, 2022). Due to this, it is of great interest to develop novel promising inhibitors targeting the CCHFV Gn/Gc mediated entry process.

Natural products contain structurally diversified and large amounts of bioactive chemicals, providing valuable sources for new drug discovery. Among the new medicines approved by the U.S. Food and Drug Administration (FDA) between 1981 and 2019, natural products or their derivatives account for 36.3% (Newman & Cragg, 2020). Previously, we have generated a library consisting of 500 pre-fractionated extracts from 100 medicinal herbs that are frequently included in traditional Chinese medicine (TCM) recipes for the use of antiviral treatment (Kang et al., 2020). In this study, we screened the TCM fractions library using a CCHFV pseudovirus (CCHFVpv)-based antiviral assay, and discovered that multi-fractions of Rhizoma Paridis exhibited CCHFV entry inhibition effects. Furthermore, the antiviral activity-guided isolation was carried out, and various saponin constituents from Rhizoma Paridis were identified as novel CCHFV inhibitors, providing a new class of natural products for the development of novel anti-CCHFV drugs in the future.

## 2 Results

### 2.1 Establishment of a CCHFV Gn/Gc based pseudovirus system for antiviral screen

**Fig. 1:**
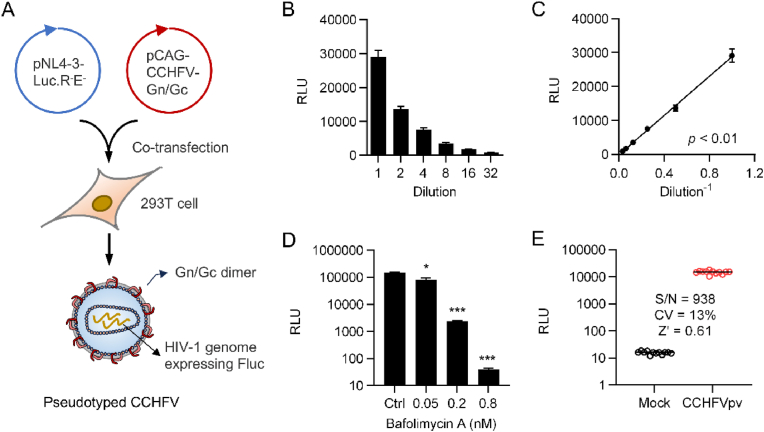
**Establishment of the pseudotyped CCHFV-based antiviral screening approach.** (A) Generation of the pseudotyped CCHFV. The CCHFV Gn/Gc-based HIV-1 pseudovirus particles (CCHFVpv) were produced by co-transfecting plasmids encoding CCHFV Gn/Gc (pCAG-CCHFV-Gn/Gc) and the HIV core plasmid (pNL4-3-Luc.R^-^E^-^). (B) Pseudoviral infectivity determination. The CCHFVpv were diluted serially and used to infect 293T cells growing in 92-well plates. At 36 h (h) post-infection (p.i.), a luciferase assay was performed and the enzymatic activity (relative light units, RLUs) was used to monitor the viral infectivity. (C) The correlation between CCHFVpv infectivity and the dose of initial infection. (D) Dose-dependent antiviral activity of Bafolimycin A against CCHFVpv. (E) Establishment of a CCHFVpv-based high-throughput antiviral screening approach. S/N, Signal to noise ratio; CV, coefficient of variation; Z′, Z value. Error bars indicate standard deviation (SD) of three independent replicates. ∗*p* < 0.05, ∗∗∗*p* < 0.001, students' *t*-test.

Operation with infectious CCHFV requires the high containment BSL-3/4 facilities, greatly hindering both the research of viral biology and the development of antiviral therapies. To alleviate the biosafety concern, we sought to develop a CCHFV Gn/Gc based human immunodeficiency virus-1 (HIV-1) pseudovirus system, a surrogate assay widely used for entry studies of highly pathogenic enveloped viruses, including CCHFV (Vasmehjani et al., 2020). Briefly, single cycle infectious pseudotyped CCHFVs (CCHFVpv) were generated by co-transfecting a plasmid expressing CCHFV Gn/Gc (pCAG-CCHFV-Gn/Gc) and an env-deficient HIV vector (pNL4-3-Luc.R^-^E^-^) into 293T producer cells (Fig. 1(A)). To test the infectivity of CCHFVpv, 293T cells were infected with serially diluted CCHFVpv. The luciferase activity of infected cells was determined 36 h post-infection (p.i.) and presented as relative luciferase units (RLUs), as shown in Fig. 1(B). The data indicates that CCHFVpv can successfully infect 293T cells and the infectivity is well correlated with the initial dose of infection (Fig. 1(C)).

To test the sensitivity of CCHFVpv to potent entry inhibitors, the target cells were treated with different concentrations of Bafilomycin A1, a well-known inhibitor of CCHFV entry process (Simon et al., 2009), for 30 min prior to incubation with the CCHFVpv. As a result, Bafilomycin A1 significantly prevented the infection of CCHFVpv in a dose dependent manner (Fig. 1(D)), suggesting that the CCHFVpv system is feasible to examine the potent inhibitory effects of test compounds on virus entry. Subsequently, the CCHFVpv system was adapted for antiviral screening. As shown in Fig. 1(E), the CCHFVpv-based entry assay can generate a signal-to-noise (S/N) ratio of as high as 938, co-efficient of variation of 13% and Z value of 0.61, providing an ideal high-throughput screening approach for novel virus entry inhibitors.

### 2.2 Discovery of Rhizoma Paridis and Bruceae fructus as potent inhibitory actives against CCHFV entry

**Fig. 2:**
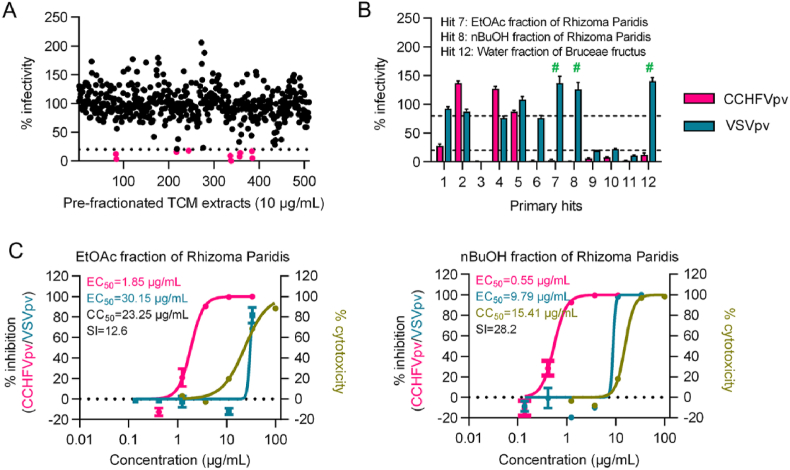
**Identification of Rhizoma Paridis fractions as potential inhibitory actives against CCHFVpv entry.** (A) Antiviral screen. A library of 500 fractions of traditional Chinese medicine was screened at 10 μg/mL against CCHFVpv, and twelve fractions that reduced viral infectivity to 20% or lower were selected as primary hit fractions. (B) Hit validation. The inhibitory effects of the primary hit fractions against CCHFVpv and VSVpv were examined in parallel to validate the antiviral activity and specificity respectively. Only the fractions that reduced CCHFVpv infectivity to <20% while showing no-inhibitory effects against VSVpv (with infectivity >80%), were confirmed as hit actives. Error bars indicate standard deviation (SD) of two independent replicates. #, confirmed hit fractions. (C) Dose dependent antiviral determination. The dose-response curves against CCHFVpv or VSVpv entry, as well as the cytotoxicity curve of the ethyl acetate (EtOAc) and N-Butanol (nBuOH) fractions derived from Rhizoma Paridis were determined. Error bars indicate standard deviation (SD) of three independent replicates.

To discover novel CCHFV entry inhibitors, we initially screened a library consisting of 500 pre-fractionated extracts from 100 TCM herbs at a concentration of 10 μg/mL using the CCHFVpv system. As shown in Fig. 2(A), 12 fractions were identified as primary hits that inhibit CCHFVpv entry by more than 80%. Then, the primary hits were subjected to a validation assay to confirm the anti-CCHFV activity. In addition, the potent inhibition of the primary hits against a VSV GP-based HIV-1 pseudovirus (VSVpv) was tested in parallel. Note that those hits shared against both CCHFVpv and VSVpv should be excluded, since they may act in a non-specific manner by suppressing luciferase activity directly, blocking the HIV-1 genome replication, or causing cytotoxicity. As a result, 3 out of the 12 hits were confirmed to possess specific inhibitory activities against CCHFV entry, including the ethyl acetate (EtOAc) and N-Butanol (nBuOH) fractions of Rhizoma Paridis, as well as the water fraction of Bruceae Fructus (Fig. 2(B)).

We chose to start with Rhizoma Paridis for further analysis. The dose-response of both EtOAc and nBuOH fractions of Rhizoma Paridis against CCHFVpv were subsequently determined, exhibiting EC_50_ values of 1.85 and 0.55 μg/mL, respectively (Fig. 2(C)). The cytotoxicities and antiviral activities against VSVpv of the Rhizoma Paridis fractions were also tested, generating CC_50_ and EC_50_ values of 23.25 and 30.15 μg/mL, respectively for the EtOAc fraction, and 15.41 and 9.79 μg/mL, respectively for the nBuOH fraction. Altogether, these data demonstrated the specificity of Rhizoma Paridis fractions for their inhibitory effects against CCHFV entry.

In addition, considering the constituents and antiviral efficacy of medicinal herbs may vary between batches, we prepared four batches of Rhizoma Paridis that were produced from different places and years, followed by comparing their anti-CCHFV inhibitory effects. As shown in Fig. S1, all four batches of Rhizoma Paridis showed comparable specific inhibition against CCHFV entry, and the actives are mainly present in the EtOAc and nBuOH fractions. These results suggest that the anti-CCHFV activity of Rhizoma Paridis is batch-independent and the active compounds appeared to be of highly polar.

### 2.3 Bioactivity guided isolation of anti-CCHFV constituents from Rhizoma Paridis

**Fig. 3:**
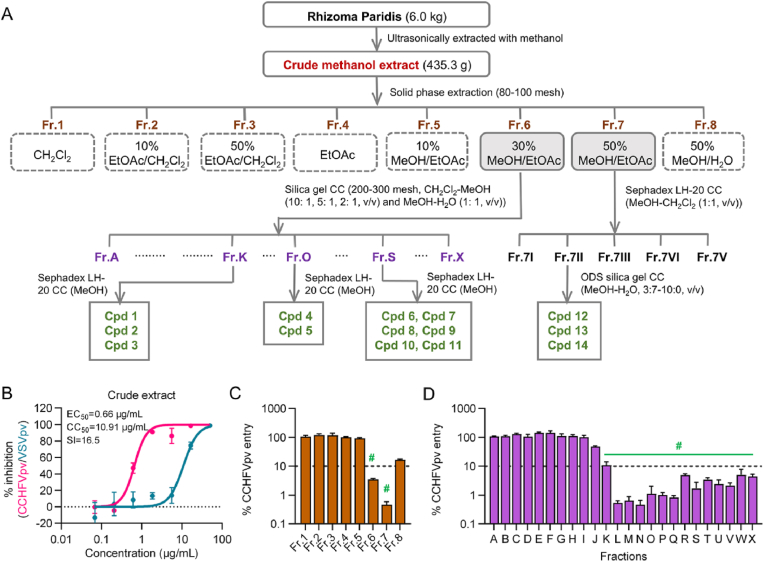
**Bioactivity-guided isolation of anti-CCHFVpv actives from Rhizoma Paridis.** (A) Procedures for the bioactivity-guided fractionation and isolation of active constituents from Rhizoma Paridis. (B) The dose-response curves of the crude Rhizoma Paridis extract against CCHFVpv as well as VSVpv. (C) The inhibitory effects of fractions 1-8 against CCHFVpv at 5 μg/mL. (D) The inhibitory effects of fractions A-X against CCHFVpv at 5 μg/mL. Error bars indicate standard deviation (SD) of two independent replicates. #, hit fractions.

To identify the precise actives of Rhizoma Paridis against CCHFV entry, a bioactivity-guided fractionation and purification process was carried out (Fig. 3(A)). Initially, a batch of Rhizoma Paridis (batch number: Yunnan-200601) was ultrasonically extracted with methanol, and the crude extract was validated for its anti-CCHFV activity with EC_50_ and CC_50_ values of 0.66 and 10.91 μg/mL, respectively (Fig. 3(B)). Notably, since the EC_50_ curve of the active Rhizoma Paridis fraction against VSVpv is almost identical to its CC_50_ curve (Fig. 2(C)), we decided to examine the CC_50_ values of Rhizoma Paridis fractions simply using the EC_50_ curves against VSVpv in the following bioactivity-guided isolation process.

Considering the liquid-liquid extraction method may induce severe emulsification, the crude methanol Rhizoma Paridis extract was next partitioned with SPE to achieve better enrichment of active components. As a result, eight distinct fractions were yielded, among which the 30% MeOH/EtOAc and 50% MeOH/EtOAc fractions exhibited significant inhibitory activity against CCHFVpv at a concentration of 5 μg/mL (Fig. 3(C)–S3 and Table 1). Consequently, further separation was carried out from these two fractions.

**Table 1:** The inhibitory effects of active Rhizoma Paridis fractions against CCHFV entry.

Fractions	EC_50_ (μg/mL)	CC_50_ (μg/mL)	SI
**Fr.6**	4.58	>50	>10.9
**Fr.7**	0.60	9.94	16.6
**Fr.K**	0.76	>50	>65.8
**Fr.L**	0.51	18.12	35.5
**Fr.M**	0.53	8.38	15.8
**Fr.N**	0.63	7.41	11.8
**Fr.O**	0.57	10.54	18.5
**Fr.P**	0.65	8.39	12.9
**Fr.Q**	0.40	6.57	16.4
**Fr.R**	1.20	23.88	19.9
**Fr.S**	1.02	50.86	49.9
**Fr.T**	2.13	32.22	15.1
**Fr.U**	1.52	38.93	25.6
**Fr.V**	1.40	27.46	19.6
**Fr.W**	2.06	43.95	21.3
**Fr.X**	1.62	42.58	26.3

The 30% MeOH/EtOAc fraction was further divided into 24 subfractions designated Fr.A − Fr.X by silica gel CC, among which Fr.K − Fr.X demonstrated potent antiviral activity (Fig. 3(D)–S3 and Table 1). Through additional purification steps involving Sephadex LH-20 CC and preparative HPLC, 11 saponins were isolated, including compounds (**1**) Polyphyllin H (Zhang et al., 2011), (**2**) Polyphyllin I (Zhang et al., 2011), (**3**) Prosapogenin A of dioscin (Hu et al., 1996), (**4**) Dioscin (Tai et al., 2018), (**5**) Gracillin (Hu et al., 1996), (**6**) Polyphyllin VII (Zhang et al., 2011), (**7**) Polyphyllin VI (Teng et al., 2019), (**8**) Polyphyllin II (Munday et al., 1993), (**9**) Polyphyllosside IV (Chen & Zhou, 1995), (**10**) Parisaponin I (Kang et al., 2007) and (**11**) Dioscoreanoside I (Tapondjou et al., 2013).

Meanwhile, the 50% MeOH/EtOAc fraction was fractionated into five subfractions designated Fr.I − Fr.V using Sephadex LH-20 CC. And 3 additional saponins were separated sequentially by ODS silica gel CC and preparative HPLC from Fr.II, including compounds (**12**) Parisyunnanoside G (Kang et al., 2012), (**13**) Parisyunnanoside K (Jiang et al., 2022) and (**14**) Parisyunnanoside H (Kang et al., 2012).

**Fig. 4:**
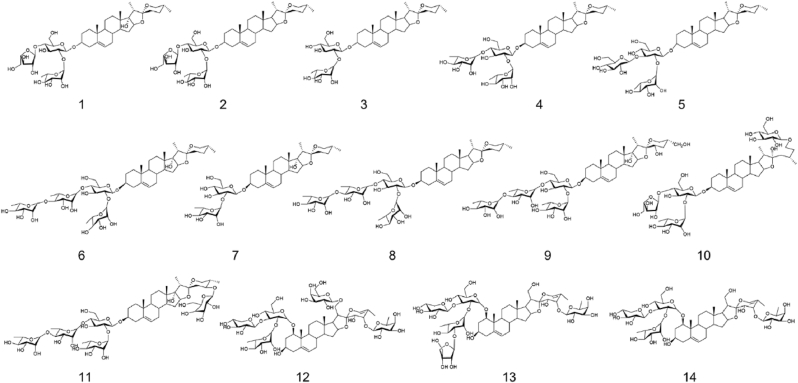
The chemical structures of the Rhizoma Paridis saponins.

The chemical structures of the isolated saponins were listed in Fig. 4. Of note, besides the saponins, several compounds of other chemical scaffolds were also isolated from the active fractions. However, these compounds showed no anti-CCHFV activity at as high as 10 μg/mL (Fig. S2).

### 2.4 Identification of Rhizoma Paridis saponins as novel CCHFV entry inhibitors

Next, the anti-CCHFV effects of the isolated Rhizoma Paridis saponins were evaluated (Fig. S4), and the EC_50_ values against CCHFVpv and VSVpv as well as the CC_50_ value of each saponin were summarized in Table 2. Almost all the saponins except compounds (**8**) Polyphyllin II and (**9**) Polyphylloside IV showed inhibitory effects against CCHFV entry, with EC_50_ and selective index (SI) values ranging from 0.53 to 5.98 μM and 4.1-20, respectively. The specificity of the anti-CCHFV activities of the saponins was also validated, as none of the compounds exhibit inhibition against VSVpv (Fig. S4, Table 2). All these together suggest that the saponins are the major active constituents for the anti-CCHFV properties of Rhizoma Paridis.

**Table 2:** The inhibitory effects of Rhizoma Paridis saponin derivatives against CCHFV entry.

Compound No.	Chemical name	EC_50_ (μM)		CC_50_ (μM)	SI
		CCHFVpv	VSVpv		
1	Polyphyllin H	0.88	>20	10.73	12.2
2	Polyphyllin I	0.85	>20	7.82	9.2
3	Prosapogenin A	5.27	>20	21.61	4.1
4	Dioscin	2.12	21.28	15.66	7.4
5	Gracillin	1.01	20.69	12.28	12.2
6	Polyphyllin VII	0.82	15.53	5.26	6.4
7	Polyphyllin VI	1.95	19.50	11.08	5.7
8	Polyphyllin II	1.12	4.66	0.64	--
9	Polyphyllosside IV	>20	>20	>20	--
10	Parisaponin I	1.24	15.83	22.41	18.1 ∗
11	Dioscoreanoside I	0.53	17.83	4.37	8.2
12	Parisyunnanoside G	1.55	16.13	30.97	20.0 ∗
13	Parisyunnanoside K	2.29	10.44	25.90	11.3
14	Parisyunnanoside H	5.98	20.82	10.47	1.75

### 2.5 Rhizoma Paridis extract and the saponin constituent exhibit antiviral activity against authentic CCHFV

**Fig. 5:**
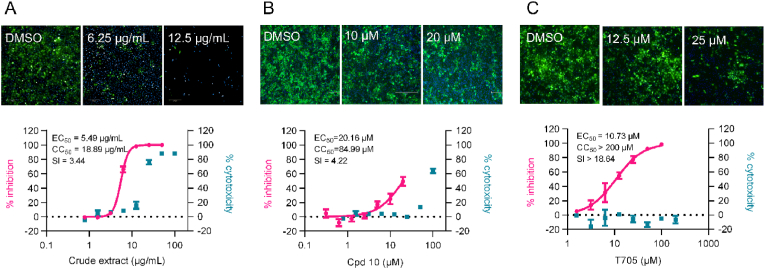
**Antiviral determination against authentic CCHFV.** The inhibitory effects of (A) the crude extract of Rhizoma Paridis, (B) compound (Cpd) 10 derived from Rhizoma Paridis, and (C) a well-known RdRp inhibitor, T705, were examined against the authentic CCHFV in HUEVC cells. Upper panels, immunofluorescence images. Lower panels, dose-response curves. Error bars indicate standard deviation (SD) of three independent replicates.

To further validate the anti-CCHFV activity of Rhizoma Paridis extract and its active components, the crude methanol extract of Rhizoma Paridis and two of the most potent active components, compound (**10**) Parisaponin I and (**12**) Parisyunnanoside G, were evaluated against authentic CCHFVs in a BSL-3 facility. As shown in Fig. 5(A), the fluorescence assay demonstrated that the crude methanol extract of Rhizoma Paridis remarkably reduced CCHFV infection at 6.25 and 12.5 μg/mL, without showing obvious cytotoxicity. The inhibition and cytotoxicity data were then fitted to a dose-response curve, generating EC_50_ and CC_50_ values of 5.49 and 18.89 μg/mL, respectively. For compounds **(10)** and **(12)**, as compound **(12)** did not show any inhibitory effect at as high as 20 μM (data not shown), compound **(10)** significantly blocked CCHFV infection at non-cytotoxic concentrations (Fig. 5(B)). The EC_50_ and CC_50_ values were calculated by dose-response curve fitting as 20.16 and 84.99 μM, respectively, generating a SI value of 4.22. Note that the positive control T705, a well-known RdRp inhibitor, showed a reasonable EC_50_ value of 10.73 and SI value of over 18.64 (Fig. 5(C)). These results clearly demonstrated the antiviral activity of Rhizoma Paridis extract and the derivative saponins against authentic CCHFV.

### 2.6 Rhizoma Paridis saponins block CCHFV entry by targeting host cells

**Fig. 6:**
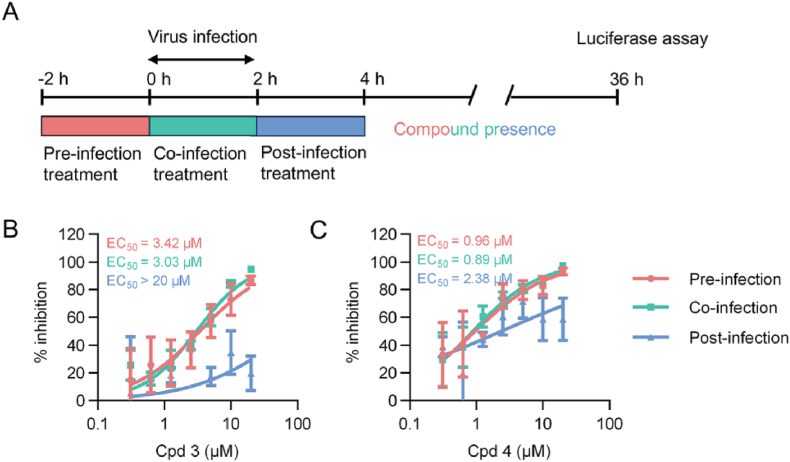
**Time-of-addition assay.** (A) The strategy diagram for the time-of-addition assay. (B and C) Dose-response curves of compound (Cpd) 3 and 4 against CCHFVpv for indicated drug adding intervals. Error bars indicate standard deviation (SD) of three independent replicates.

To better understand the mechanism of action underlying the inhibition of CCHFV entry by Rhizoma Paridis saponins, a time-of-addition experiment was performed. As shown in Fig. 6(A), 293T cells were inoculated with CCHFVpv and incubated for 2 h, then the inoculum was removed and fresh medium was added. The timepoint of virus inoculation was set as 0 h. For antiviral treatment, the active saponins, compounds **(3)** or **(4)** were added at intervals of −2-0 h (pre-infection), 0-2 h (co-infection), and 2-4 h (post-infection, p.i.), separately. DMSO treatment was carried out in parallel as negative control. At 36 h p.i., the viral infectivity was determined. As shown in Fig. 6(B) and (C), both pre- and co-infection treatment with indicated compounds markedly blocked CCHFVpv infection, exhibiting almost identical dose-response curves. While upon post-infection treatment, the dose-response curve shifted to the right, suggesting reduced activities. These results imply that Rhizoma Paridis saponins act against CCHFV entry by interfering with the host factors, albeit the precise mechanism of action remains elusive and further investigation is needed in future.

## 3 Discussion

In the present study, we established a pseudotyped CCHFV system, providing a BSL-2 compliant surrogate assay to study the entry process of the highly pathogenic pathogen. Subsequently, this assay was adapted for antiviral screening, and identified Rhizoma Paridis and Bruceae Fructus from a 500 pre-purified TCM fractions library to possess potent CCHFV entry inhibitory effects. Next, we focused on Rhizoma Paridis for further analysis. Using an antiviral activity guided isolation strategy, a class of saponins were obtained, characterized, and validated as novel CCHFV entry inhibitors. Moreover, the antiviral activity of Rhizoma Paridis crude extract and the derivative saponins was validated using authentic CCHFV in a BSL-3 containment library.

Saponins are a large family of amphiphilic glycosides of steroids and triterpenes found in terrestrial plants as well as some marine organisms. These natural products exhibit a plethora of pharmacological properties, including antiviral, anti-inflammatory, anticancer, antifungal, antimicrobial, antioxidant, and immunomodulatory effects (Xu & Yu, 2021). Since ancient times, many kinds of saponins have served as key ingredients in folk medicines, making them attractive candidates for innovative drug design and development (Xu & Yu, 2021). In the present study, we demonstrated that a range of Rhizoma Paridis saponins showed anti-CCHFV activities. It can be anticipated that a combination of these Rhizoma Paridis saponins may perform better than a single one, probably by additive or even synergistic effects. Indeed, our data have demonstrated the anti-CCHFV activity of the crude Rhizoma Paridis extract. Moreover, the isolation of a single saponin from natural sources is usually considered to be challenging due to the microheterogeneity, while the crude Rhizoma Paridis extract or total Rhizoma Paridis saponins can be much easier prepared.

According to the mechanism of action, antiviral agents can be divided into two classes: the direct-acting antivirals (DAAs), which act upon viral components, and the host-targeting antivirals (HTAs), which interfere with host factors that are pivotal for virus replication. Generally, HTAs possess several unique advantages over DAAs (Du et al., 2024). First, HTAs rarely induce the emergence of drug resistance. Second, HTAs possess potential broad-spectrum antiviral activity against heterogenous virus strains, or even diverse genotypes or genera. Interestingly, our data have demonstrated that the Rhizoma Paridis saponins exert their anti-CCHFV activity by interfering with host factors, although the precise targets of action have not been clarified in the present study. In the future, it will be interesting to identify the potential targets and molecular mechanisms underlying the anti-CCHFV property of Rhizoma Paridis saponins.

The virus entry of CCHFV is a comprehensive process, not only the viral surface proteins Gn/Gc but also numerous host factors contribute to this process. To date, a number of host factors have been reported for their critical role during CCHFV entry, such as the low-density lipoprotein receptor (LDL-R) and apolipoprotein E (apoE) which act as viral entry receptors or co-receptors, the phosphatidylinositol 3-kinase (PI3K) pathway which is related to the multivesicular bodies (MVBs) formation, as well as the proteins that are involved in the endosomal sorting complex required for transport (ESCRT) (Ritter et al., 2024; Shtanko et al., 2014; Xu et al., 2024). However, the comprehensive map of viral-host interactions during CCHFV entry remains largely unexplored. Considering the Rhizoma Paridis saponins block virus entry by targeting host factors, it can be anticipated that elucidating the precise targets of action may greatly advance our understanding of the virus entry mechanism, and imply promising strategies for the development of novel CCHFV inhibitors.

In summary, our study identified the herbal medicine Rhizoma Paridis and its saponin derivatives as potential therapeutic agents against CCHFV infections, either as a therapy or as a complement to conventional pharmacological strategies.

## 4 Methods and materials

### 4.1 Cells and viruses

Human embryonic kidney cells (HEK293T) and human umbilical vein endothelial cells (HUVEC) were cultured in Dulbecco's modified Eagle medium (DMEM) supplemented with 10% fetal bovine serum (FBS) and 1% penicillin/streptomycin (10 000 U/mL). All cells were cultured at 37 °C under 5% CO_2_.

CCHFV-IbAr10200-eGFP (National Virus Resource Center of China, ID: CSTR:16533.06; IVCAS6.9295) was propagated and stored as previously reported (Liu et al., 2024). All experiments involving the authentic CCHFV were conducted in the National High-level Biosafety Laboratory, Wuhan, Chinese Academy of Sciences (CAS), with BSL-3 facilities.

### 4.2 TCM fractions library and medicinal herbs

The library of 500 pre-fractionated antiviral TCMs was prepared and stored as previously described (Kang et al., 2020). The various batches of Rhizoma Paridis were purchased from Anhui Helin Traditional Chinese Medicine Decoction Piece Technology Co., Ltd. (Anhui, China; batch number: Yunnan-200601, Yunnan-201702, Yunnan-201807, Yunnan-201909, Guizhou-201903) and was authenticated by Professor Lingchuan Xu (College of Pharmacy, Shandong University of Traditional Chinese Medicine). A voucher specimen (CL-2020) has been deposited in the pharmaceutical chemistry laboratory of Shandong University of Traditional Chinese Medicine.

### 4.3 Plasmids and reagents

The coding sequence of CCHFV-IbAr10200 GPC (NC_005300) was cloned into pCAGGS to obtain pCAG-CCHFV-Gn/Gc. The plasmid expressing vesicular stomatitis virus (VSV) G (pCAG-VSVG) and the replication-defective HIV vector (pNL4-3-Luc.R^-^E^-^) were generated as previously described (Chen et al., 2024).

Sephadex LH-20 and ODS C18 silica gel were purchased from GE Healthcare Bio-Sciences AB (Uppsala, Sweden). Silica gel of 80 - 100 and precoated TLC sheets of silica gel GF254 were purchased from Qingdao Marine Chemical Inc. (Qingdao, China). HPLC-gradient methanol was supplied by CINC High Purity Solvents (Shanghai, China) Co., Ltd. Dichloromethane (CH_2_Cl_2_), methanol (MeOH) and ethyl acetate (EtOAc) were purchased from Tianjin Fuyu Chemical Co., Ltd (Tianjin, China). The ultra-pure water was prepared in our laboratory using a Rephile Direct-pure UP system (Rephile Bioscience Ltd., Shanghai, China).

### 4.4 Generation of pseudoviruses

The pseudoviruses were generated as previously described with slight modifications (Du et al., 2021). Briefly, the CCHFV Gn/Gc and VSV G-based HIV pseudoviruses termed CCHFVpv and VSVpv respectively, were produced by transient co-transfection of a corresponding GP encoding plasmid (pCAG-CCHFV-Gn/Gc and pCAG-VSVG, separately) and the HIV core plasmid (pNL4-3-Luc.R^-^E^-^) into 293T cells using Lipofectamine 2000 (Invitrogen, CA, USA), according to the manufacturer's instructions. The supernatants were collected and filtered through 0.45 μm pore size filter (Nalgene, Rochester, NY, USA) at 24 h (h) post-transfection. The pseudovirus stocks were stored at 4 °C before use.

### 4.5 Pseudovirus based antiviral assay

To examine the infectivity of CCHFVpv or VSVpv, the pseudovirus stocks were used to infect 293T cells grown in 96-well plates for 36 h. Then, the cells were harvested and subjected to a luciferase assay. The luciferase activity (relative light units, RLUs) was used to indicate the virus infectivity. For antiviral determination, the infection was performed in the presence of TCM fractions at indicated concentrations for antiviral screening or increasing concentrations of the test extract or compound for dose-response analysis.

### 4.6 Bioactivity guided isolation of Rhizoma Paridis

Isolation of active compounds from Rhizoma Paridis was guided by the inhibitory effects against CCHFVpv. After each step of fractionation, only the bioactive fractions were selected for further purification.

Air-dried Rhizoma Paridis (6.0 kg) were finely chopped and subjected to ultrasonic extraction in methanol (6L × 5, 40 min each), with the resulting extract concentrated under reduced pressure (50 °C) to yield a brownish residue (435.3 g). The residue was then fractionated by segmented solid phase extraction (SPE) with silica gel (80-100 mesh) as the adsorbent, and eluted with CH_2_Cl_2_, 10% EtOAc in CH_2_Cl_2_ (10% EtOAc/CH_2_Cl_2_), 50% EtOAc in CH_2_Cl_2_ (50% EtOAc/CH_2_Cl_2_), EtOAc, 10% MeOH in EtOAc (10% MeOH/EtOAc), 30% MeOH in EtOAc (30% MeOH/EtOAc), 50% MeOH in EtOAc (50% MeOH/EtOAc) and 50% MeOH in H_2_O (50% MeOH/H_2_O) successively to obtain 8 fractions (Fr.1 to Fr.8).

The 30% MeOH/EtOAc fraction (Fr.6, 117.0 g) was subjected to silica gel (200-300 mesh) column chromatography (CC), using gradient solvent systems of CH_2_Cl_2_-MeOH (10:1, 5:1, 2:1, v/v) and MeOH-H_2_O (1:1, v/v), to afford 24 fractions (Fr.A − Fr.X) based on TLC analysis. Fr.K (4.1 g) was then chromatographed on a Sephadex LH-20 column and eluted with MeOH to give 17 subfractions (Fr.K-1 to Fr.K-17). Subsequent chromatography of Fr.K-6 over preparative HPLC with 83% to 91% MeOH in H_2_O as mobile phase led to the isolation of compound **1** (8.1 mg), **2** (2.3 mg) and **3** (2.1 mg). Fr.O (4.4 g) was purified by Sephadex LH-20 CC (MeOH) and preparative HPLC (96% MeOH in H_2_O) to produce compound **4** (3.5 mg) and **5** (4.4 mg). Fr.S (10.8 g) was chromatographed on a Sephadex LH-20 column with MeOH as eluent, and 28 subfractions (Fr.S-1 to Fr.S-28) were obtained. Fr.S-11 to Fr.S-12 were merged and then purified by preparative HPLC (86% to 94% MeOH in H_2_O) to yield compound **6** (11.8 mg), **7** (2.3 mg), **8** (5.6 mg) and **9** (3.2 mg). Compound **10** (6.7 mg) was obtained from Fr.S-13 by preparative HPLC (30% MeCN in H_2_O containing 0.1% formic acid). Fr.S-14 was purified by preparative HPLC (80% MeOH in H_2_O) to obtain compound **11** (7.0 mg).

The 50% MeOH/EtOAc fraction (Fr.7, 57.6 g) was fractionated by a Sephadex LH-20 column eluted with MeOH-CH_2_Cl_2_ (1:1, v/v) to produce 5 fractions (Fr.7I − Fr.7V). Fr.7II (3.6 g) was subjected to ODS silica gel (166.0 g), eluting with MeOH-H_2_O (3:7-10:0, v/v) to produce four subfractions (Fr.7II-1 − Fr.7II-4). Fr.-7II-2 was further purified by preparative HPLC. Water (A) - Methanol (B) system was used as the mobile phase in gradient mode as follows: 0-15 min, methanol was kept at 48%; 15-35 min, methanol was kept at 53%; 35-55 min, methanol was kept at 57%; 55-80 min, methanol was kept at 100%. Compound **12** (7.0 mg), **13** (10.2 mg) and **14** (5.6 mg) were yielded at 35 min, 46 min and 48 min successively.

Nuclear magnetic resonance (NMR) spectra were recorded on a Bruker Ascend 600 MHz AVANCE III NMR spectrometers (Bruker, Germany) with TMS as an internal standard. The HR-ESI-MS spectra were taken on an Agilent 6540 UHD quadrupole time-of-flight mass spectrometry (Q-TOF MS, Agilent Technologies). Preparative HPLC was performed on FLEXA-HP purification system (Tianjin Agela Technologies Company, China) equipped with a UV detector and a hplcone 8C18-100AA (20 mm × 250 mm, 8 μm) column.

### 4.7 Infectious virus assay

To evaluate the antiviral activity of anti-pseudovirus positive TCM extractions or derived compounds against authentic viruses, CCHFV- IbAr10200-eGFP infection of HUVEC in the presence of compounds was performed following the approved standard operation procedures of BSL-3 laboratory at Wuhan Institute of Virology, Chinese Academy of Sciences. HUVEC were seeded in 96-well plate (2 × 10^4^ cells/well), and were infected with CCHFV- IbAr10200-eGFP (MOI = 0.1) on the following day in the presence of compounds with indicated concentration. Three replicates were conducted for each treatment, and DMSO incubations were used as negative controls. After 1 h incubation, cell supernatants were discarded and replaced with fresh medium with compounds. At 48 h p.i., cells were fixed with 4% paraformaldehyde and EGFP fluorescence was quantified with a High Content Imaging System Operetta CLS (PerkinElmer, NY, USA) to illustrate the viral infection level. The inhibitory effect of compounds on virus infection was normalized to that of DMSO control. A dose-response curve was generated with GraphPad Prism 8.0.

### 4.8 Time-of-addition experiment

To illustrate the mechanism of action for Rhizoma Paridis saponins against CCHFV entry, a time-of-addition assay was performed. Briefly, the pseudotyped CCHFV was used to inoculate 293T cells (0 h) and incubated at 37 °C for 2 h. Then, the inoculum was removed and the cells were washed with PBS three times. Fresh culture medium was added, followed by incubation at 37 °C for 36 h before luciferase assay. For pre-, co- and post-infection treatment, increasing concentrations of test saponins were added at −2-0 h, 0-2 h and 2-4 h intervals, respectively. For all experiments, DMSO treatment was performed in parallel as negative control.

## CRediT authorship contribution statement

**Ruikun Du:** Writing – review & editing, Writing – original draft, Supervision, Investigation, Funding acquisition, Formal analysis, Conceptualization. **Chen Liu:** Writing – original draft, Investigation, Formal analysis. **Xi Wang:** Writing – review & editing, Investigation. **Huiying Hou:** Investigation. **Xu Wang:** Methodology, Investigation. **Zhaoyu Chen:** Investigation. **Jazmin Galvan Achi:** Writing – review & editing. **Qinghua Cui:** Investigation, Funding acquisition. **Lijun Rong:** Writing – review & editing, Supervision, Conceptualization. **Manli Wang:** Writing – review & editing, Supervision, Conceptualization. **Rong Rong:** Writing – review & editing, Supervision, Conceptualization.

## Declaration of competing interest

The authors declare that they have no known competing financial interests or personal relationships that could have appeared to influence the work reported in this paper.

## Data Availability

All data are available in the manuscript or supporting materials.
