# Supplementary material for: Isolation of Rhizoma Paridis saponins as novel entry inhibitors of Crimean-Congo hemorrhagic fever virus
Source: Cell Insight. 2026 Apr 28;5(3):100328. doi: 10.1016/j.cellin.2026.100328 (PMC13187606; doi:10.1016/j.cellin.2026.100328)
Supplement: Multimedia component 1 [file mmc1.docx]

*Supplementary data*

**Isolation of Rhizoma Paridis saponins as novel entry inhibitors of Crimean-Congo haemorrhagic fever virus**

Ruikun Du ^1,#,*^, Chen Liu ^2,#^, Xi Wang ^3,#^, Huiying Hou ^1,#^, Xu Wang ^2^, Zhaoyu Chen ^2^, [Jazmin Galvan Achi](https://pubmed.ncbi.nlm.nih.gov/?sort=date&term=Achi+JG&cauthor_id=38899934) ^4^, Qinghua Cui ^1^, Lijun Rong ^4,*^, Manli Wang ^3,*^, Rong Rong ^2,*^

^1^ Qingdao Academy of Chinese Medical Sciences, Shandong University of Traditional Chinese Medicine, Qingdao 266041, China

^2^ College of pharmacy, Shandong University of Traditional Chinese Medicine, Jinan 250355, China

^3^ State Key Laboratory of Virology, Wuhan Institute of Virology, Center for Biosafety Mega-Science, Chinese Academy of Sciences, 430071, Wuhan, China

^4^ Department of Microbiology and Immunology, University of Illinois at Chicago, Chicago, Illinois 60612, USA

# These authors contributed equally to this work.

* Correspondence: [ruikun@sdutcm.edu.cn](mailto:ruikun@sdutcm.edu.cn) (Ruikun Du); [lijun@uic.edu](mailto:lijun@uic.edu) (Lijun Rong); wangml@wh.iov.cn (Manli Wang); [rosierong@163.com](mailto:rosierong@163.com) (Rong Rong)

**
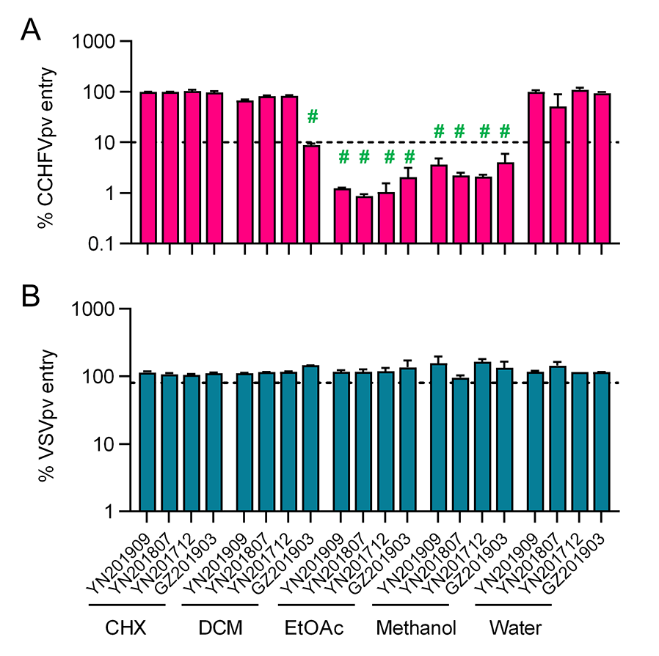
**

**Figure S1** The inhibitory effects of the fractions derived from different batches of Rhizoma Paridis against CCHFVpv (A) and VSVpv (B) at 5 μg/mL. Error bars indicate standard deviation (SD) of two independent replicates. #, hit fractions.

**
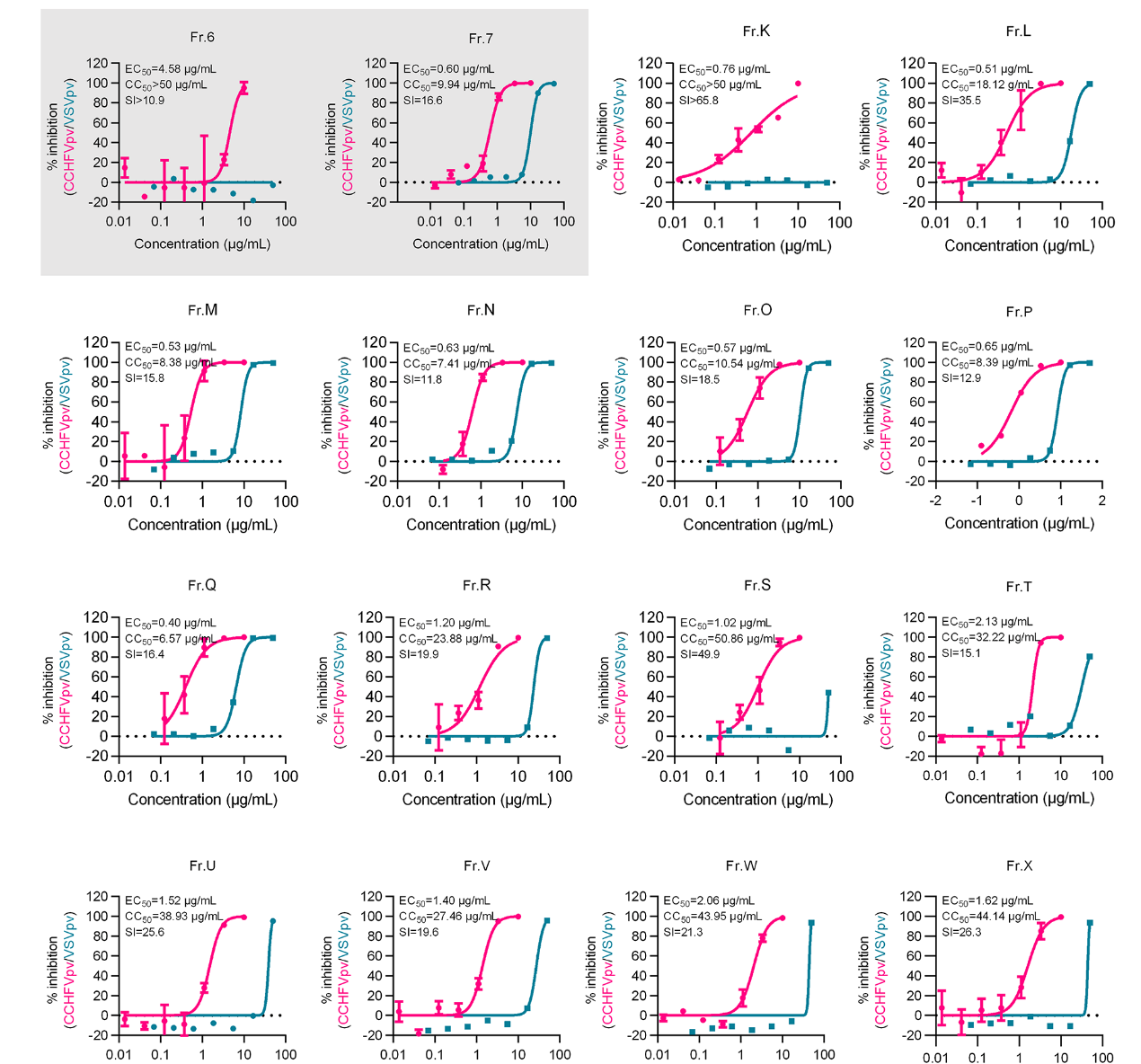
Figure S2 Dose-response curves of hit fractions.**

**
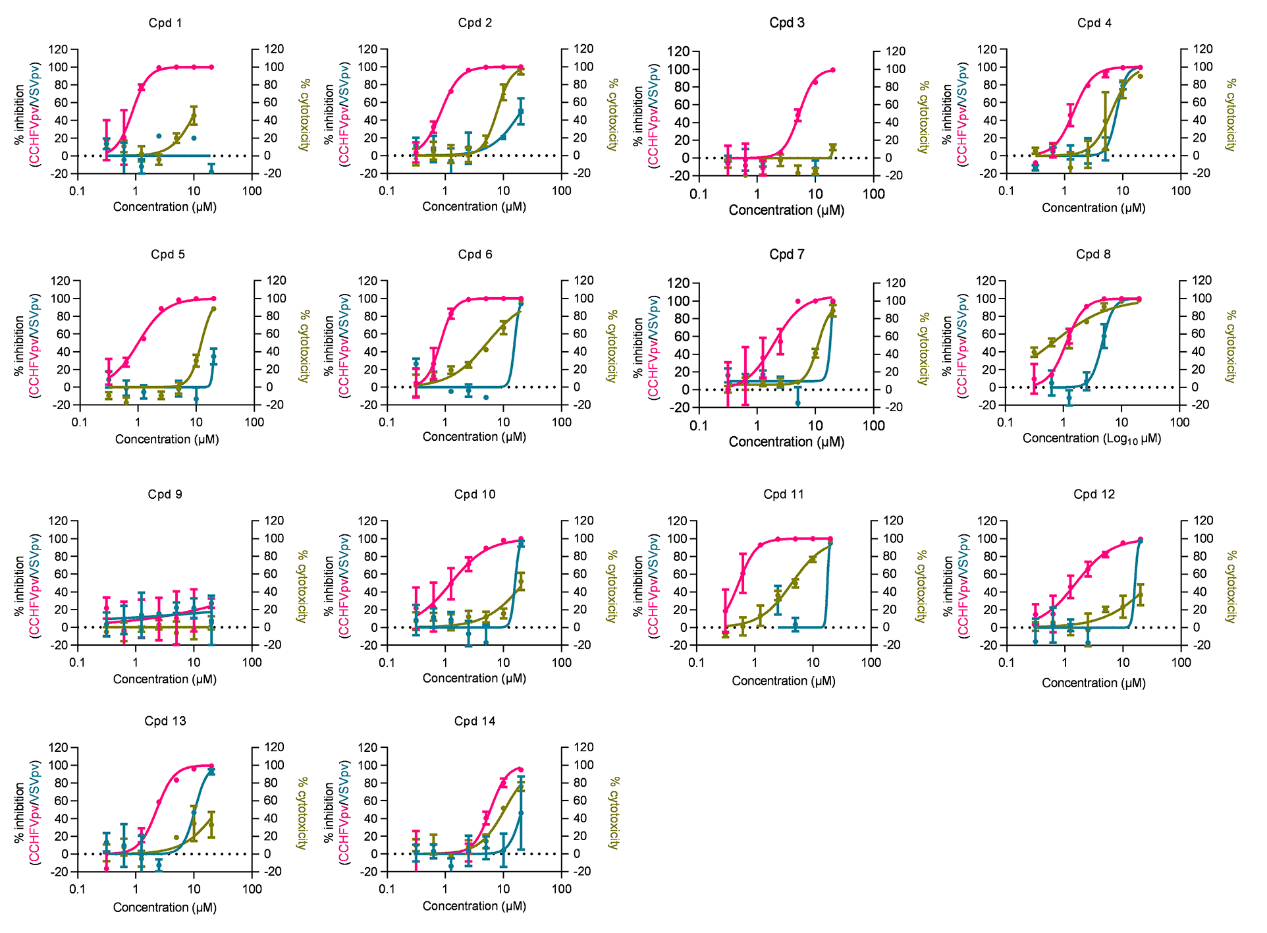
Figure S3 Dose-response curves of the Rhizoma Paridis saponins.**

**
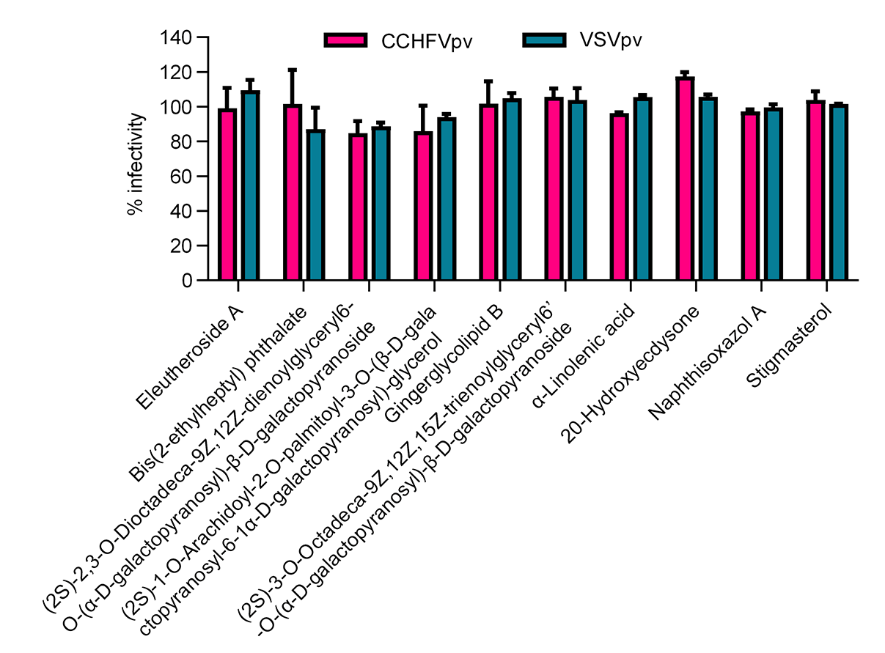
**

**Figure S4.** Antiviral determination of the constituents derived from active fractions of Rhizoma Paridis other than saponins against CCHFVpv and VSVpv at 5 μg/mL.
